# Human skeletal muscle mitochondrial dynamics in relation to oxidative capacity and insulin sensitivity

**DOI:** 10.1007/s00125-020-05335-w

**Published:** 2020-11-30

**Authors:** Alexandre Houzelle, Johanna A. Jörgensen, Gert Schaart, Sabine Daemen, Nynke van Polanen, Ciarán E. Fealy, Matthijs K. C. Hesselink, Patrick Schrauwen, Joris Hoeks

**Affiliations:** Department of Nutrition and Movement Sciences, NUTRIM School of Nutrition and Translational Research in Metabolism, Maastricht University Medical Center, Maastricht, the Netherlands

**Keywords:** Fission, FIS1, Fusion, HSP60, Insulin sensitivity, Mitochondria, OPA1, Oxidative phosphorylation, Skeletal muscle

## Abstract

**Aims/hypothesis:**

Mitochondria operate in networks, adapting to external stresses and changes in cellular metabolic demand and are subject to various quality control mechanisms. On the basis of these traits, we here hypothesise that the regulation of mitochondrial networks in skeletal muscle is hampered in humans with compromised oxidative capacity and insulin sensitivity.

**Methods:**

In a cross-sectional design, we compared four groups of participants (selected from previous studies) ranging in aerobic capacity and insulin sensitivity, i.e. participants with type 2 diabetes (*n* = 11), obese participants without diabetes (*n* = 12), lean individuals (*n* = 10) and endurance-trained athletes (*n* = 12); basal, overnight fasted muscle biopsies were newly analysed for the current study and we compared the levels of essential mitochondrial dynamics and quality control regulatory proteins in skeletal muscle tissue.

**Results:**

Type 2 diabetes patients and obese participants were older than lean participants and athletes (58.6 ± 4.0 and 56.7 ± 7.2 vs 21.8 ± 2.5 and 25.1 ± 4.3 years, *p* < 0.001, respectively) and displayed a higher BMI (32.4 ± 3.7 and 31.0 ± 3.7 vs 22.1 ± 1.8 and 21.0 ± 1.5 kg/m^2^, *p* < 0.001, respectively) than lean individuals and endurance-trained athletes. Fission protein 1 (FIS1) and optic atrophy protein 1 (OPA1) protein content was highest in muscle from athletes and lowest in participants with type 2 diabetes and obesity, respectively (FIS1: 1.86 ± 0.79 vs 0.79 ± 0.51 AU, *p* = 0.002; and OPA1: 1.55 ± 0.64 vs 0.76 ± 0.52 AU, *p* = 0.014), which coincided with mitochondrial network fragmentation in individuals with type 2 diabetes, as assessed by confocal microscopy in a subset of type 2 diabetes patients vs endurance-trained athletes (*n* = 6). Furthermore, lean individuals and athletes displayed a mitonuclear protein balance that was different from obese participants and those with type 2 diabetes. Mitonuclear protein balance also associated with heat shock protein 60 (HSP60) protein levels, which were higher in athletes when compared with participants with obesity (*p* = 0.048) and type 2 diabetes (*p* = 0.002), indicative for activation of the mitochondrial unfolded protein response. Finally, OPA1, FIS1 and HSP60 correlated positively with aerobic capacity (*r* = 0.48, *p* = 0.0001; *r* = 0.55, *p* < 0.001 and *r* = 0.61, *p* < 0.0001, respectively) and insulin sensitivity (*r* = 0.40, *p* = 0.008; *r* = 0.44, *p* = 0.003 and *r* = 0.48, *p* = 0.001, respectively).

**Conclusions/interpretation:**

Collectively, our data suggest that mitochondrial dynamics and quality control in skeletal muscle are linked to oxidative capacity in humans, which may play a role in the maintenance of muscle insulin sensitivity.

**Clinical Trial registry:**

numbers NCT00943059, NCT01298375 and NL1888

**Figure:**
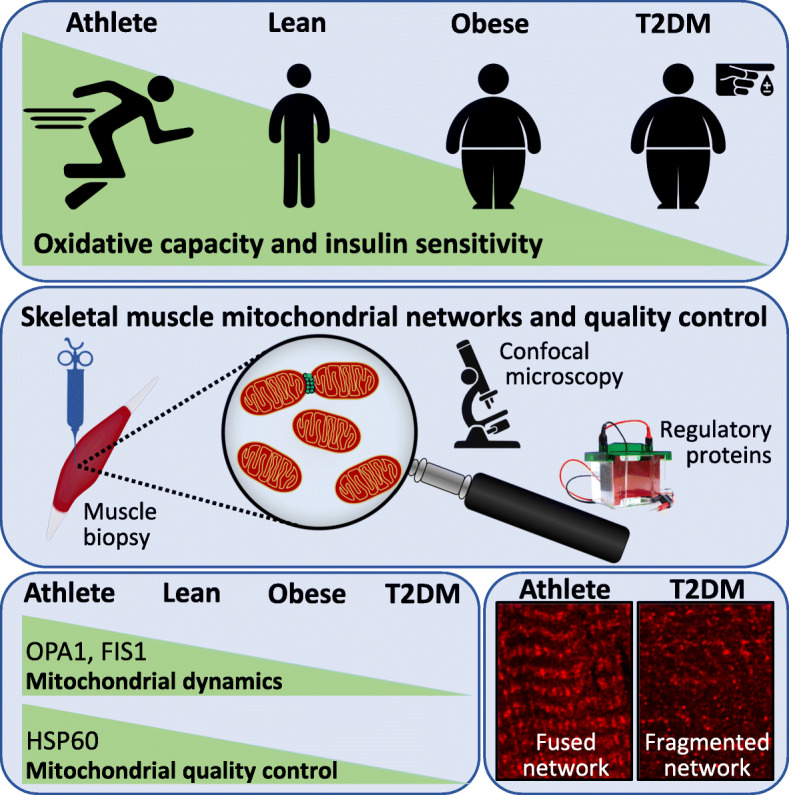
Graphical abstract

**Supplementary Information:**

The online version contains peer-reviewed but unedited supplementary material available at 10.1007/s00125-020-05335-w.

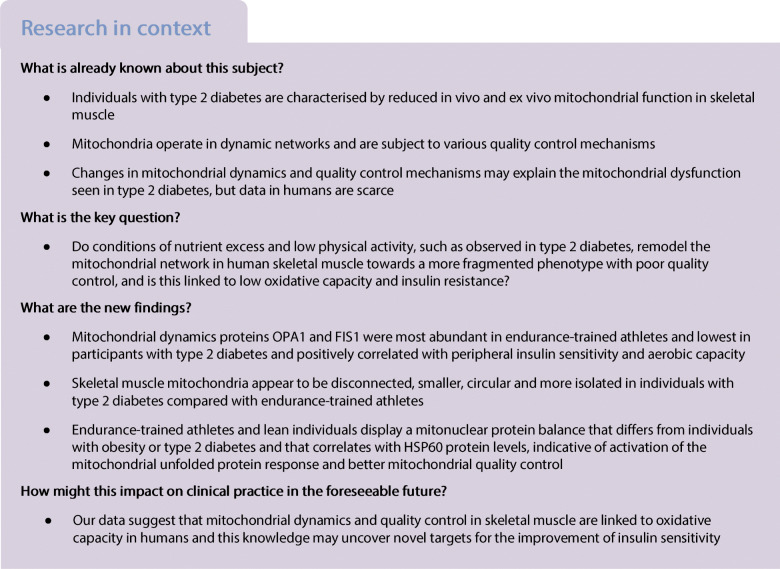



## Introduction

Over the past decades, a large number of studies have linked a reduced mitochondrial capacity to the aetiology of skeletal muscle insulin resistance and type 2 diabetes (reviewed in [1–3]). Despite the numerous reports of mitochondrial dysfunction in type 2 diabetes, the hypothesis that a reduced mitochondrial capacity is the primary cause of insulin resistance has been substantially challenged over the years [4–6]. Nonetheless, even if secondary to insulin resistance, mitochondrial defects may aggravate metabolic impairments in skeletal muscle and further reduce insulin sensitivity.

Mitochondria operate in tightly regulated, dynamic networks [7] that are subject to several quality control mechanisms to maintain a healthy mitochondrial function, in a process called mitochondrial dynamics. This process is defined by the perpetual fusion and fission of mitochondria (or mitochondrial parts) within the mitochondrial network, which allows the integration of newly formed mitochondria as well as the removal of damaged or senescent mitochondria via mitophagy [8]. Furthermore, mitochondrial dynamics have been suggested to play an important role in the regulation of nutrient utilisation and energy expenditure [9]. Mitochondrial fission is mainly regulated by dynamin-related protein 1 (DRP1) and fission protein 1 (FIS1) whereas fusion is controlled by the two mitofusin proteins (MFN1/2) and by the optic atrophy 1 (OPA1) protein, responsible for the fusion of outer and inner mitochondrial membranes, respectively [8]. A disturbed balance between fusion and fission, based on gene expression levels of involved proteins in skeletal muscle, has been identified in ageing, obesity and type 2 diabetes [10, 11]. In addition, it has been shown that fusion and fission proteins respond to lifestyle interventions such as caloric restriction, physical activity [12, 13] and following Roux-en-Y gastric bypass (RYGB) surgery in rats [14]. Few studies have examined the mitochondrial network in human skeletal muscle [15, 16], especially in relation to insulin resistance and mild obesity. Kristensen et al [17] showed that the mitochondrial network in muscle from morbidly obese participants with type 2 diabetes (42 kg/m^2^) was more fragmented compared with a lean reference group. Following RYGB surgery, the mitochondrial network appeared more organised and interconnected [17].

Mitochondrial dynamics are also tied to mitochondrial quality control, which is ensured by the mitochondrial unfolded protein response (mtUPR) [18], i.e. the induction of proteases and chaperones in response to oxidative or proteotoxic stress [19] and mitophagy, i.e. the selective removal of damaged mitochondria by autophagosomes [20]. Interestingly, changes in mitochondrial quality control mechanisms have also been linked to insulin resistance and mitochondrial function [21, 22], albeit not in skeletal muscle and not in humans.

Here, we hypothesise that under conditions of nutrient excess and low physical activity, such as observed in type 2 diabetes, the mitochondrial network is remodelled towards a more fragmented phenotype with poor quality control, which is subsequently linked to low oxidative capacity and insulin resistance. Information on mitochondrial dynamics and quality control in human skeletal muscle from insulin-sensitive vs insulin-resistant individuals is scarce and the few studies available do not link these changes to phenotypical outcomes related to oxidative capacity and insulin sensitivity. Therefore, we here compared essential mitochondrial dynamics and quality control regulatory proteins in skeletal muscle biopsies of well-characterised individuals ranging in insulin sensitivity and (mitochondrial) oxidative capacity, i.e. participants with type 2 diabetes, obese individuals, lean individuals and endurance-trained athletes. Next to measuring protein content of players in mitochondrial dynamics we also imaged the mitochondrial network at high resolution in a subset of skeletal muscle biopsies to actually map putative changes in network connectivity.

## Methods

### Study population

The participants included in this study were derived from previous studies all performed at Maastricht University, approved by the institutional medical ethics committee and registered at ClinicalTrials.gov (NCT00943059 and NCT01298375) and in the Dutch trial register (https://www.trialregister.nl/trial/1888) [23–26]. All participants (*n* = 45) gave their written consent in accordance with the declaration of Helsinki.

From these studies (all male individuals), four metabolically distinct groups were selected, i.e. individuals with type 2 diabetes (*n* = 11), obese individuals without diabetes (*n* = 12), lean individuals (*n* = 10) and endurance-trained athletes (*n* = 12) and basal, overnight fasted muscle biopsies were newly analysed for the current study. Participants were selected based on the availability of muscle tissue as well as the presence of previously collected data on peripheral insulin sensitivity and maximal aerobic capacity, to allow correlation analysis.

Lean participants were included if their $\dot{V}{\mathrm{O}}_{2\max }$ was below 45 ml min^−1^ [kg body weight]^−1^. Endurance-trained athletes were included if they had a $\dot{\ V}{\mathrm{O}}_{2\max }$ above 55 ml min^−1^ [kg body weight]^−1^. Body composition was either determined via hydrostatic weighing according to the method of Siri [27] or via dual x-ray absorptiometry (DEXA), and $\dot{V}{\mathrm{O}}_{2\max }$ was determined using an incremental cycling test essentially according to Hawley and Noakes [28]. $\dot{V}{\mathrm{O}}_{2\max }$ was expressed relative to both body weight and fat-free mass (FFM) and normalised to FFM when reported in the figures.

### Hyperinsulinaemic–euglycaemic clamp

Insulin sensitivity was determined using a 40 mU m^−2^ min^−1^ hyperinsulinaemic–euglycaemic clamp as described previously [29, 30]. All participants were fasted overnight before the clamp and received a standardised meal on the evening prior the clamp. Participants were instructed to avoid physical exercise for 3 days before the glucose clamp. Glucose infusion rates (GIR) were expressed relative to both body weight and FFM and normalised to FFM when reported in the figures.

### Plasma analyses

Prior to the commencement of the clamp, a fasted blood sample was obtained from an antecubital vein and collected in an EDTA-containing vacutainer. Fasting glucose and NEFA levels were determined in plasma using enzymatic assays on Cobas Bio Fara and Mira analyzers (Roche, Basel, Switzerland).

### Skeletal muscle biopsies

In the morning, following an overnight fast, a biopsy was taken under local anaesthesia from the vastus lateralis muscle prior to any measurement. The muscle biopsy was immediately freed from blood and other non-muscle material, frozen immediately in melting isopentane cooled with liquid nitrogen and stored at −80°C.

### Protein extraction and western blot analysis

Western blot analyses were performed in whole lysates of human skeletal muscle tissue. Groups were randomly coded to blind the analyses but to also allow representation of all groups on each blot. Briefly, ~10 mg of muscle tissue was homogenised and dissolved in RIPA buffer and total protein content was determined using a colorimetric bicinchoninic acid (BCA) assay. Subsequently, 10 μg of protein, according to the BCA assay, was loaded on gradient mini-protean Bolt 4–12% Bis-Tris Plus gels (Invitrogen, Carlsbad, CA, USA). Proteins were then transferred to nitrocellulose using a Trans-blot turbo transfer system (Bio-Rad, Hercules, CA, USA). Similar loading was checked on nitrocellulose membranes by performing Revert Staining (Li-Cor, Lincoln, NE, USA), which was then used for normalisation. For every blotting experiment, a 4-point standard curve was included from a pooled tissue homogenate, to ensure inter-blot reproducibility and to determine linear protein detection range. The following antibodies and dilutions were used in this study: MFN1 (Ab-57602, 1:1000, Abcam, Cambridge, UK), MFN2 (sc-100560, 1:1000, Santa Cruz Biotechnology, Dallas, TX, USA), OPA1 (BD-612606, 1:2500, BD Biosciences, San Jose, CA, USA), FIS1 (sc-98900, 1:1000, Santa Cruz Biotechnology), DRP1 (CS-8570, 1:1000, Cell Signaling Technology, Beverly, MA, USA), microtubule-associated protein light chain 3B (LC3B; Sigma-L7543, 1:1000, Sigma Aldrich, Zwijndrecht, the Netherlands), phosphatase and tensin homologue (PTEN)-induced putative kinase 1 (PINK1; sc-33796, 1:2000, Santa Cruz Biotechnology), heat shock protein 60 (HSP60; sc-59567, 1:5000, Santa Cruz Biotechnology), voltage-dependent anion-selective channel 1 (VDAC1; sc-58649, 1:5000, Santa Cruz Biotechnology), cytochrome C oxidase subunit 4I1 (COX4I1; 4850, 1:10000, Cell Signaling Technology) and total oxidative phosphorylation (OXPHOS; ms-601, 1:5000, Mitosciences, Eugene, OR, USA), a cocktail containing five monoclonal antibodies against NDUFB8 (Complex I), SDHB (Complex II), UQCRC2 (Complex III), cytochrome C oxidase subunit II (COX II; Complex IV) and ATP5A (Complex V). Membranes were then incubated with an appropriate fluorescently tagged secondary antibody and proteins of interest were detected and quantified using an Odyssey Near Infrared Imager (Li-Cor). In cases where proteins appear as a doublet (i.e. MFN2, OPA1, DRP1, VDAC1, LC3B), both bands were analysed and the sum of the bands was quantified.

### Mitochondrial staining and confocal imaging

Muscle cryosections of 5 μm were cut and mounted on a glass slide as previously described [31]. Immunofluorescence analyses were performed on 12 participants (6 type 2 diabetes patients vs 6 endurance-trained athletes), selected according to the lowest and highest GIR during the glucose clamp, respectively. To minimise variability in staining, individual sections of type 2 diabetes patients and athletes were mounted in a paired fashion on the same glass slide. Sections were incubated for 60 min with primary antibodies against caveolin as a marker protein of the plasma membrane to detect individual muscle fibres (610421, BD Biosciences, Franklin Lakes, NJ, USA), translocase of the outer mitochondrial membrane (TOMM20) as a mitochondrial marker protein (Ab186734, Abcam, Cambridge, UK) and myosin heavy chain type I (MHC1) to detect muscle fibre typology (A4.840, Developmental Studies Hybridoma Bank, Iowa City, IA, USA). Thereafter, sections were incubated for 2 h with appropriate secondary antibodies labelled with AlexaFluor 405, 488 and 555 (Invitrogen-ThermoFisher, Groningen, the Netherlands). Confocal image acquisition of longitudinally cut muscle fibres was performed on a Leica TCS SP8 STED microscope in confocal mode. Images were deconvolved using Huygens Professional Software (Scientific Volume Imaging, Hilversum, the Netherlands). Sections with apparent freezing or tissue damage were discarded for analysis.

### Statistical analyses

Participant characteristics and protein expression were analysed with one-way ANOVA with Tukey’s multiple comparisons test. Relationships between protein expression and metabolic variables such as GIR and maximal aerobic capacity were assessed using Pearson correlation analysis. Values are expressed as mean ± SD. A *p* value below 0.05 was considered statistically significant.

## Results

### Participant characteristics

Participant characteristics are summarised in Table 1. By design, obese individuals and type 2 diabetes patients were significantly older and had a higher body weight, BMI and percentage body fat in comparison with lean individuals and endurance-trained athletes (all *p* < 0.001). The type 2 diabetes patients and the obese individuals without diabetes on the one hand and the lean individuals and the endurance-trained athletes on the other, were similar in age and BMI. Furthermore, type 2 diabetic participants displayed the lowest $\dot{V}{\mathrm{O}}_{2\max }$ whereas athletes showed superior maximal aerobic capacity, also in comparison with lean individuals (*p* < 0.001).

**Table 1:** Participant characteristics

Characteristic	T2DM *	Obese ^†^	Lean ^‡^	Athletes ^§^
*n*	11	12	10	12
Age (years)	58.6 ± 4.0 ‡‡‡, §§§	56.7 ± 7.2 ‡‡‡, §§§	21.8 ± 2.5 ***, †††	25.1 ± 4.3 ***, †††
Body weight (kg)	101.7 ± 13.4 ‡‡‡, §§§	94.1 ± 13.8 ‡‡‡, §§§	72.0 ± 6.6 ***, †††	70.3 ± 7.4 ***, †††
Height (m)	1.77 ± 0.08	1.74 ± 0.08 §	1.80 ± 0.05	1.83 ± 0.07 †
BMI (kg/m^2^)	32.4 ± 3.7 ‡‡‡, §§§	31.0 ± 3.7 ‡‡‡, §§§	22.1 ± 1.8 ***, †††	21.0 ± 1.5 ***, †††
Fat (%)	34.4 ± 5.8 ‡‡‡, §§§	34.7 ± 7.0 ‡‡‡, §§§	17.9 ± 3.7 ***, †††	12.8 ± 2.1 ***, †††
FFM (kg)	66.2 ± 7.0	61.2 ± 9.2	59.1 ± 6.1	61.3 ± 6.3
$\dot{V}{\mathrm{O}}_{2\max }$ (ml min^−1^ [kg_BW_]^−1^)	24.6 ± 4.1 ‡‡‡, §§§	27.8 ± 4.3 ‡‡‡, §§§	41.5 ± 2.0 ***, †††, §§§	60.0 ± 4.1 ***, †††, ‡‡‡
$\dot{V}{\mathrm{O}}_{2\max }$ (ml min^−1^ kg_FFM_^−1^)	37.3 ± 4.5 ‡‡‡, §§§	42.8 ± 6.9 ‡‡, §§§	50.5 ± 3.8 ***, ††, §§§	68.7 ± 4.4 ***, †††, ‡‡‡
GIR (μmol kg_BW_^−1^ min^−1^)	15.0 ± 7.2 ‡‡‡, §§§	26.6 ± 8.6 ‡‡‡, §§§	55.5 ± 8.3 ***, †††, §§§	76.5 ± 16.0 ***, †††, ‡‡‡
GIR (μmol kg_FFM_^−1^ min^−1^)	22.9 ± 11.1 †, ‡‡‡, §§§	40.5 ± 12.0 *, ‡‡‡, §§§	68.0 ± 12.4 ***, †††, §§	87.7 ± 18.6 ***, †††, ‡‡
Blood glucose (mmol/l)	7.7 ± 1.4 †††, ‡‡‡, §§§	5.6 ± 0.4 ***	5.2 ± 0.3 ***	5.1 ± 0.3 ***
NEFA (mmol/l)	0.59 ± 0.17	0.69 ± 0.48	0.57 ± 0.21	0.38 ± 0.15
Plasma insulin (pmol/l)	145.1 ± 60.1 ‡‡, §§	151.0 ± 92.0 ‡‡, §§§	58.5 ± 17.3 **, ††	48.5 ± 17.3 **, †††

Values presented are mean ± SD

Significance is indicated with *, †, ‡ and § representing changes compared with T2DM, obese, lean and athletic participants, respectively, and with 1, 2 and 3 symbols representing *p*<0.05, *p*<0.01 and *p*<0.001, respectively. Statistical significance was determined by one-way ANOVA followed by Tukey’s post hoc test

BW, body weight; T2DM, type 2 diabetic group

Inherent to their condition, the participants with type 2 diabetes were characterised by elevated fasting plasma glucose levels (*p* < 0.001 vs all other groups) and the lowest insulin sensitivity, as exemplified by the GIR during the clamp. Endurance-trained athletes displayed the highest insulin sensitivity, which was significantly higher than all other groups (all *p* < 0.001). Although plasma insulin levels were similar between participants with obesity and type 2 diabetes, the levels observed in these groups were significantly higher compared with both lean and endurance-trained individuals (both *p* < 0.01).

### Athletes present the highest levels of the fusion protein OPA1 and the fission protein FIS1

**Fig. 1:**
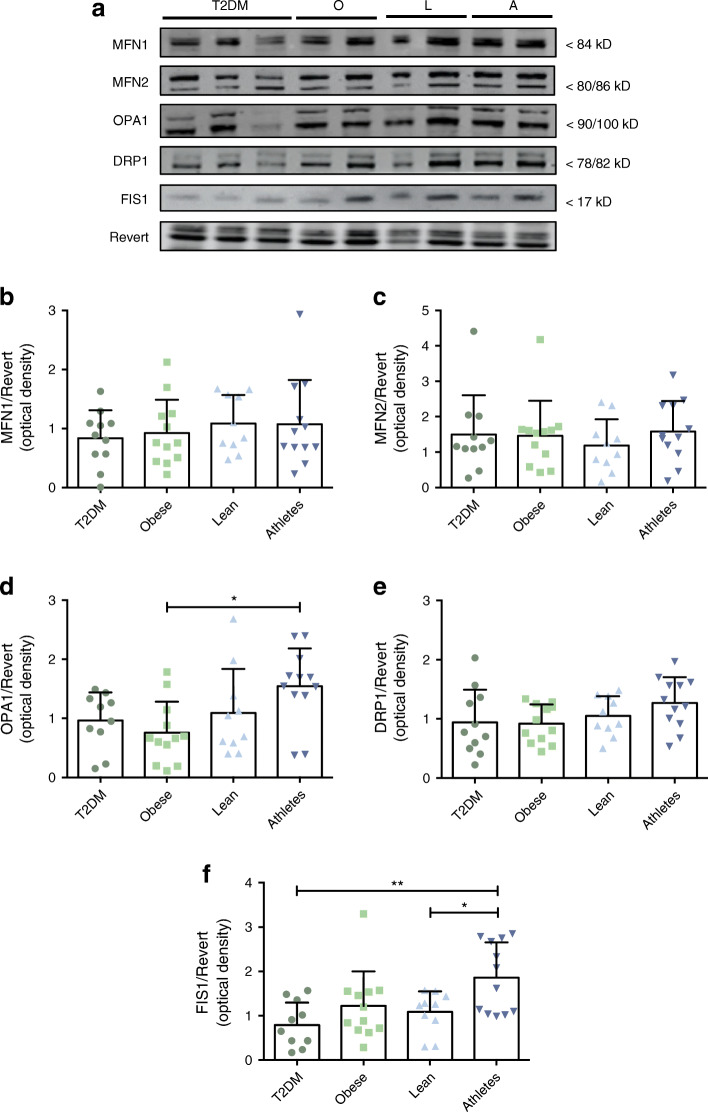
Type 2 diabetic individuals show lower expression of proteins involved in mitochondrial dynamics. Representative western blots (**a**) and associated quantifications (**b**–**f**) of mitochondrial dynamics regulatory proteins (MFN1, MFN2, OPA1, DRP1 and FIS1) in human skeletal muscle biopsies. T2DM, participants with type 2 diabetes (*n* = 11); O, obese individuals without type 2 diabetes (*n*=12); L, lean individuals (*n*=10); A, endurance-trained athletes (*n*=12). Values are normalised to Revert staining and expressed as mean ± SD. *p<0.05, **p<0.01

To investigate if and how mitochondrial dynamics relate to oxidative capacity and muscle insulin sensitivity in humans, we first investigated the content of proteins involved in mitochondrial fusion such as MFN1, MFN2 and OPA1. While no differences between groups were detected in the protein levels of MFN1/2 (Fig. 1a–c), OPA1 levels showed a differential expression pattern between groups as determined by one-way ANOVA (*p* = 0.02). Tukey’s post hoc test revealed that the difference between endurance-trained athletes, presenting the highest OPA1 protein expression, and the obese non-diabetic individuals, with the lowest OPA1 expression, was statistically significant (1.55 ± 0.64 vs 0.76 ± 0.52 AU, Fig. 1a,d; *p* = 0.014).

Next, we examined the content of proteins regulating skeletal muscle mitochondrial fission across the four phenotypically distinct participant groups. Whereas protein content of DRP1 (Fig. 1a,e), a GTPase responsible for the fission of the mitochondrial membrane, was similar across all groups, we observed a significant difference between groups (one-way ANOVA: *p* = 0.003) in FIS1 protein levels, a mitochondria-bound protein involved in mitochondrial fission and mitophagy (Fig. 1a,f). Tukey’s post hoc analysis revealed that the difference between the endurance-trained athletes and both lean (1.86 ± 0.79 vs 1.08 ± 0.46 AU; *p* = 0.04) and type 2 diabetic (1.86 ± 0.79 vs 0.79 ± 0.51 AU; *p* = 0.002) participants was statistically significant, with the athletes showing the highest protein levels (Fig. 1f). Together with the data on OPA1, these data may indicate that both the fusion and fission process is augmented in endurance-trained athletes.

### Human skeletal muscle mitochondrial network morphology is different in athletes vs type 2 diabetes patients

**Fig. 2:**
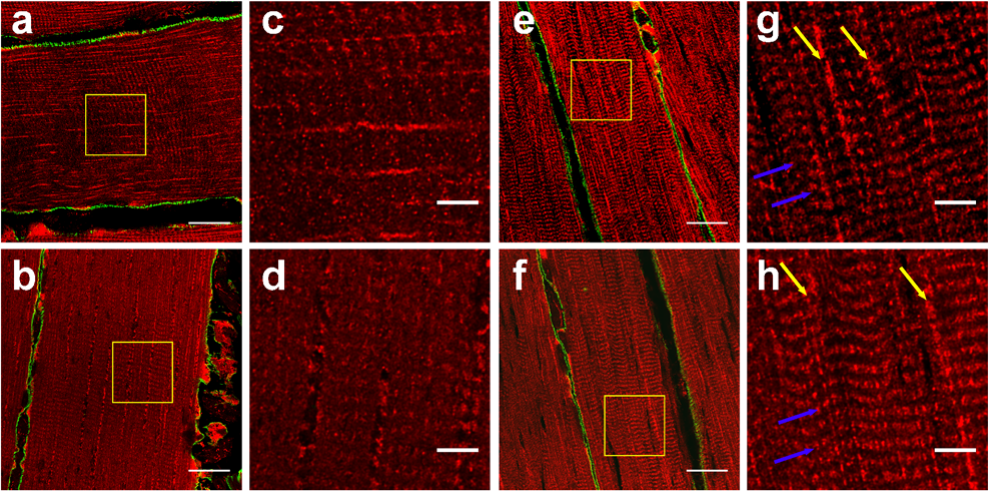
Type 2 diabetic individuals display a more fragmented skeletal muscle mitochondrial network. Representative confocal images of the mitochondrial network in type I muscle fibres. The cellular membrane was stained in green using laminin as a marker, in red the mitochondrial network is visualised using TOMM20 as a marker. In the overview images (**a**, **b** and **e**, **f**) scale bar, 20 μm; in the zoomed images (**c**, **d** and **g**, **h**) scale bar, 5 μm. (**a**–**d**) Representative overview and zoomed images of two separate individuals with type 2 diabetes. (**e**–**h**) Representative overview and zoomed images of two individual trained athletes. Note the longitudinal pattern of interconnected mitochondria alongside the myofibrils (yellow arrows) and the cross-striated pattern reflecting mitochondria near the Z-line (blue arrows) in the athletes whereas this pattern is virtually absent in the type 2 diabetic participants and the networks present a more punctate dot-like pattern, reflecting disconnected mitochondria

To investigate if the differential expression of the mitochondrial dynamics regulatory proteins coincided with an altered morphological status of the mitochondrial network, we selected six athletes and six type 2 diabetes patients with the highest and lowest GIR per kg FFM during the glucose clamp (mean GIR: 100.5 ± 17.7 μmol kg_FFM_^−1^ min^−1^, *n* = 6 vs 15.0 ± 3.0 μmol kg_FFM_^−1^ min^−1^, *n* = 6, respectively) and performed detailed confocal microscopy imaging, using the mitochondrial marker protein TOMM20. Especially in type I muscle fibres, qualitative analysis revealed a perceptible difference in mitochondrial morphology, pointing towards a more fragmented mitochondrial network with more isolated, circular mitochondria in the biopsies from patients with type 2 diabetes compared with endurance-trained athletes (Fig. 2). In type II muscle fibres, this difference in mitochondrial network morphology was less pronounced (ESM Fig. 1).

### Protein expression of skeletal muscle mitochondrial complexes

**Fig. 3:**
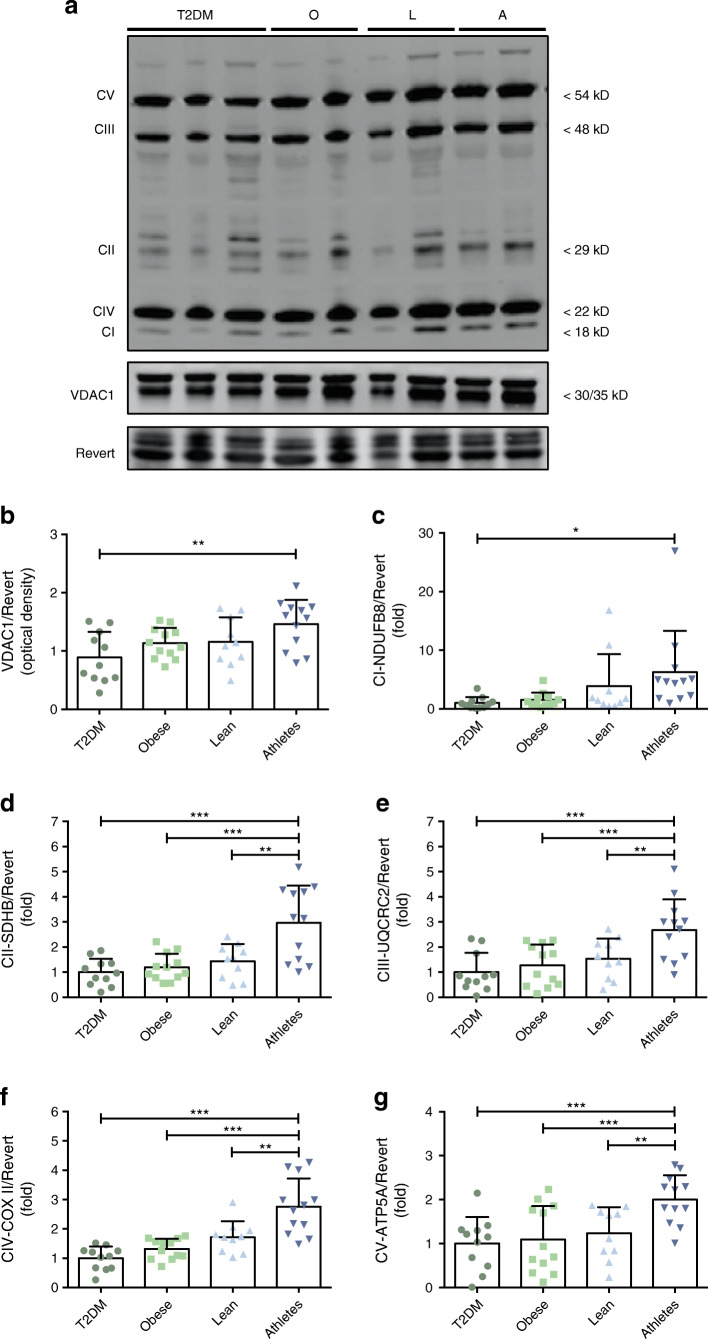
Type 2 diabetic individuals show lower mitochondrial content. Representative blots (**a**) and associated quantifications (**b**–**g**) of mitochondrial density markers VDAC1 and OXPHOS complex I (CI-NDUFB8), complex II (CII-SDHB), complex III (CIII-UQCRC2), complex IV (CIV-COX II) and complex V (CV-ATP5A) in human skeletal muscle. T2DM, participants with type 2 diabetes (*n*=11); O, obese individuals without type 2 diabetes (*n*=12); L, lean individuals (*n*=10); A, endurance-trained athletes (*n*=12). Values are normalised to Revert staining and expressed as mean ± SD. OXPHOS complexes are expressed as fold change, with T2DM set to 1. *p<0.05, **p<0.01, ***p<0.001

**Fig. 4:**
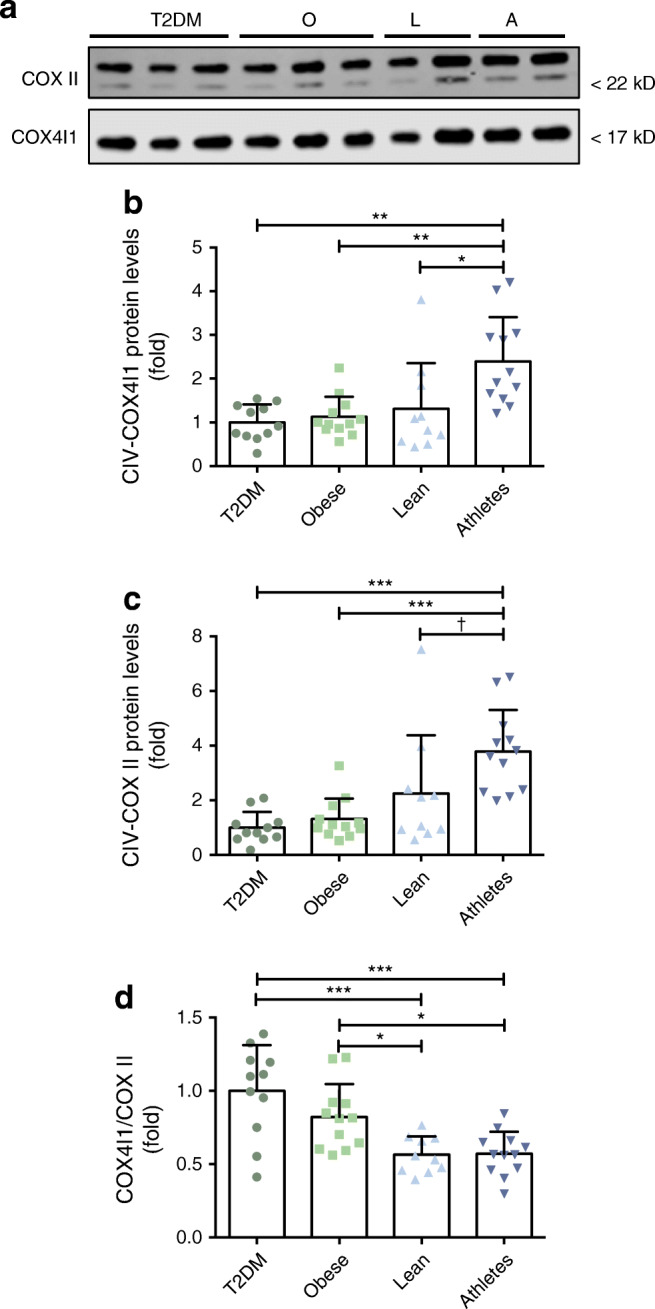
Mitonuclear protein balance differs in lean individuals and athletes vs obese and type 2 diabetic participants. Representative blots (**a**) and associated quantifications of (**b**) the nuclear-encoded complex IV (CIV) subunit COX4I1, (**c**) the mtDNA-encoded complex IV subunit COX II and (**d**) mitonuclear protein balance (COX4I1/COX II) in human skeletal muscle. Both COX4I1 and COX II were detected on the same blots. T2DM, participants with type 2 diabetes (*n*=11); O, obese individuals without type 2 diabetes (*n*=12); L, lean individuals (*n*=10); A, endurance-trained athletes (*n*=12). Values are expressed as mean fold change ± SD, with T2DM set to 1. *p<0.05, **p<0.01, ***p<0.001, ^†^*p*=0.06

Subsequently, we assessed the mitochondrial porin VDAC1 as well as structural subunits of the five electron transport chain complexes via western blot quantification, as markers for mitochondrial content. Inherent to their respective phenotypes, all of these proteins were significantly different between groups (ANOVA: VDAC1 *p* = 0.01; Complex I *p* = 0.03; Complex II *p* < 0.001; Complex III *p* < 0.001; Complex IV *p* < 0.001 and Complex V *p* = 0.002), with the endurance-trained athletes displaying the highest levels of the mitochondrial density markers, compared with the other groups (Fig. 3a–g). A disturbed balance between the nuclear DNA-encoded and mitochondrial DNA (mtDNA)-encoded OXPHOS proteins, independent of mitochondrial content, has previously been associated with the activation of mitochondrial quality control mechanisms such as the mtUPR [32, 33]. To investigate this mitonuclear protein balance, we measured the ratio between nuclear DNA-encoded (cytochrome C oxidase subunit 4I1, COX4I1, Fig. 4a,b) and mtDNA-encoded (COX II, Fig. 4a,c) subunits within complex IV of the respiratory chain and found that the ratio between these complex IV subunits was significantly different between groups (ANOVA *p* < 0.001). Tukey’s post hoc analysis revealed a statistically significant difference in mitonuclear protein balance between type 2 diabetes patients and obese individuals on the one hand and lean and endurance-trained athletes on the other hand (Fig. 4d).

### Mitochondrial stress sensor protein HSP60 shows highest expression levels in endurance-trained athletes

**Fig. 5:**
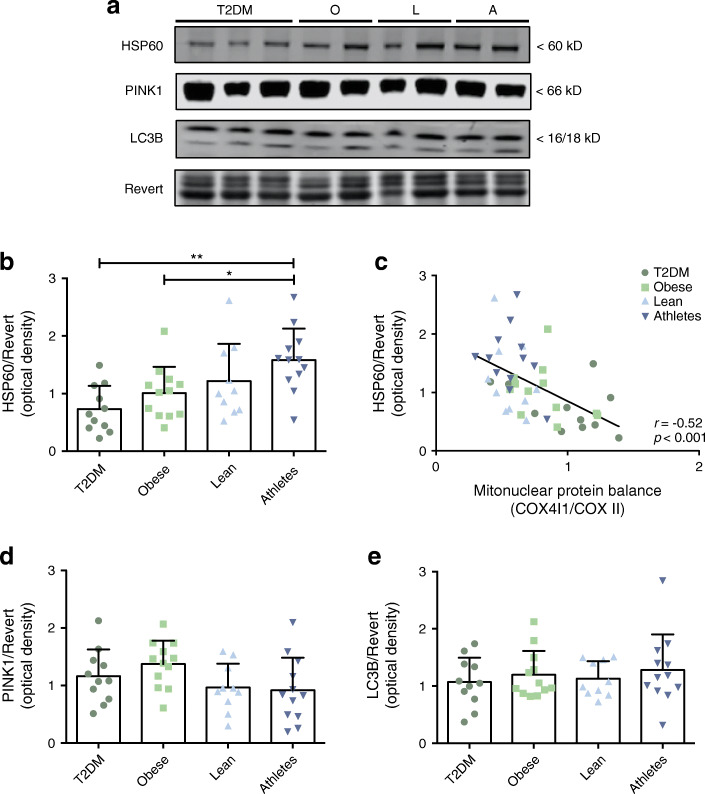
Endurance-trained athletes show highest protein levels of the mitochondrial chaperone HSP60. Representative western blot analysis (**a**) and quantification of (**b**) mitochondrial chaperone HSP60 and (**d**, **e**) mito-/autophagy-related proteins in human skeletal muscle biopsies. (**c**) Mitonuclear protein balance (COX4I1/COX II) is associated with HSP60 protein levels. T2DM, participants with type 2 diabetes (*n*=11); O, obese individuals without type 2 diabetes (*n*=12); L, lean individuals (*n*=10); A, endurance-trained athletes (*n*=12). Values are normalised to Revert staining and expressed as mean ± SD. *p<0.05, **p<0.01. Correlations are computed with Pearson’s *r*

We next investigated whether the mitonuclear protein balance was linked to the activation of the mtUPR. In line with the observed differences in mitonuclear protein balance, HSP60 protein level (Fig. 5a,b), the main chaperone protein involved in mtUPR, was significantly different across groups (ANOVA: *p* = 0.003) and displayed ~2.2- and ~1.6-fold higher levels in endurance-trained athletes compared with type 2 diabetic individuals (*p* = 0.002) and obese non-diabetic individuals (*p* = 0.048), respectively. In addition, we observed a significant correlation between the mitonuclear protein balance (i.e. COX4I1/COX II) and HSP60 protein levels (*r* = −0.52; *p* < 0.001; Fig. 5c).

We also measured protein expression of PINK1 and LC3B, proteins involved in the initiation of mitophagy and the formation of autophagosomes respectively, but the levels of these proteins were similar across all groups (Fig. 5d,e). Together, these data suggest that the mtUPR, but not selective mitophagy, is activated in endurance-trained athletes.

### Mitochondrial dynamics regulatory proteins correlate with in vivo oxidative capacity and insulin sensitivity

**Fig. 6:**
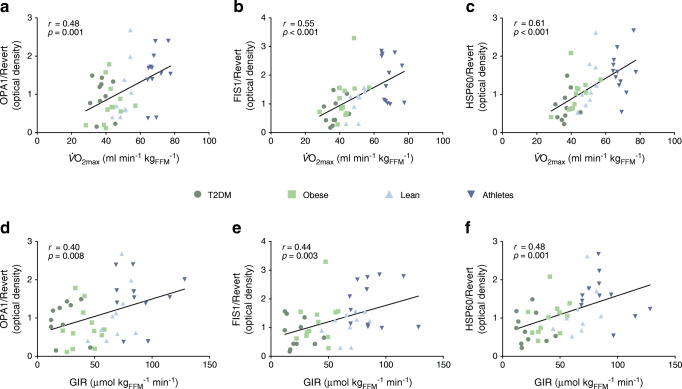
Proteins involved in mitochondrial dynamics and quality control positively correlate with metabolic variables in human skeletal muscle. Association between protein expression levels of OPA1, FIS1 and HSP60, normalised to Revert staining, with $\dot{V}{\mathrm{O}}_{2\max }$ (**a**–**c**) and insulin sensitivity (**d**–**f**). Correlations are computed with Pearson’s *r*

Finally, we investigated whether the assessed proteins, involved in the regulation of mitochondrial dynamics and mtUPR, correlated with the in vivo oxidative capacity and insulin sensitivity of the participants. Indeed, OPA1, FIS1 and HSP60 protein levels (Fig. 6) positively correlated with both $\dot{V}{\mathrm{O}}_{2\max }$ (*r* = 0.48, *p* = 0.001; *r* = 0.55, *p* < 0.001 and *r* = 0.61, *p* < 0.001, respectively) and the GIR during the clamp (*r* = 0.40, *p* = 0.008; *r* = 0.44, *p* = 0.003 and *r* = 0.48, *p* = 0.001, respectively). In addition, $\dot{V}{\mathrm{O}}_{2\max }$ and GIR were positively correlated (*r* = 0.86, *p* < 0.001, ESM Fig. 2).

## Discussion

In this cross-sectional study, we aimed to investigate players involved in the regulation of the (quality of the) mitochondrial network in skeletal muscle, in both young and older, lean and overweight/obese individuals ranging widely in both insulin sensitivity and (mitochondrial) oxidative capacity. We showed that mitochondrial dynamics regulatory proteins OPA1 and FIS1 were highest in endurance-trained athletes and lowest in obese and type 2 diabetic individuals, respectively. Confocal imaging revealed that skeletal muscle mitochondria appear to be disconnected, smaller, circular and more isolated in type 2 diabetic participants compared with endurance-trained athletes, suggesting mitochondrial fragmentation in people with type 2 diabetes.

Furthermore, we demonstrated that lean individuals and endurance-trained athletes display a mitonuclear protein balance that differs from people with obesity or type 2 diabetes. This mitonuclear protein balance also correlated to HSP60 protein levels, which were highest in athletes, indicative of the activation of the mtUPR and possibly a better mitochondrial quality control. Finally, correlation analysis showed that OPA, FIS1 and HSP60 protein expression positively correlated to peripheral insulin sensitivity and aerobic capacity.

Previously, it has been shown that a 16-week high-fat diet in mice did not change any of the mitochondrial fusion regulatory proteins in skeletal muscle, but did show an increase in the number of smaller mitochondria [34]. In another study in mice, it was demonstrated that a high-fat diet intervention induced a decrease in skeletal muscle MFN2 and OPA1 protein expression, which was restored upon 4-week exercise training [35]. Finally, in humans with type 2 diabetes it was shown that protein levels of both MFN2 and OPA1 were reduced in skeletal muscle compared with obese control individuals [36]. Collectively, these findings, including our finding that OPA1 had highest expression in endurance-trained athletes, indicate that mitochondrial fusion in skeletal muscle is positively related to mitochondrial oxidative capacity and insulin sensitivity. This notion is in line with observations that exercise training interventions increased OPA1, but not MFN2, in obese individuals [13, 37], indicating that a high aerobic capacity associates with elevated/high OPA1 levels.

We also observed that people with type 2 diabetes are characterised by low protein levels of FIS1, in comparison with endurance-trained athletes (Fig. 1). Although FIS1 is believed to be one of the DRP1 adaptor proteins, recent evidence suggests that FIS1 may instead regulate mitochondrial morphology by inhibition of fusion [38]. In addition, several reports have suggested that FIS1 may be important for mitochondrial quality control by stimulating mitophagy [39, 40]. In this context, it is possible that the higher FIS1 expression in athletes facilitates the fine tuning of mitophagy rather than an indication of increased fission. Surprisingly, DRP1, the primary actor in mitochondrial fission, was not significantly different across the four participant groups. Multiple reports suggest an increase in mitochondrial fission in skeletal muscle in obesity [17] and type 2 diabetes in humans [13, 17] and rodent models of obesity and diabetes [34, 41, 42]. Few previous studies have investigated FIS1 and DRP1 protein levels in human skeletal muscle. Joseph et al [36] reported unchanged FIS1 and DRP1 levels in type 2 diabetes patients compared with control individuals, while showing decreased MFN2 and OPA1. Kristensen et al [17] showed increased MFN2 and DRP1 protein expression in morbidly obese individuals relative to lean individuals, with no difference in FIS1 or OPA1. The discrepancies between these studies may partly be explained by the fact that the latter study normalised protein content to citrate synthase activity—a marker of mitochondrial density.

To establish if the differences in regulatory proteins coincided with changes in the morphological status of the mitochondrial network, we performed detailed confocal microscopy imaging and found that diabetes patients display a more fragmented network compared with endurance-trained athletes. This was predominantly visible in type I muscle fibres, possibly due to the fact that type I muscle fibres have been shown to display a different mitochondrial topology compared with type II fibres, with mitochondria appearing more elongated and ‘string-like’ [43]. Thus, the connectivity of the mitochondria in type II fibres is less prominent, which complicates the qualitative assessment of mitochondrial network integrity in athletes vs people with type 2 diabetes. Other studies in human skeletal muscle have shown, by electron/confocal microscopy, that individuals with (morbid) obesity and/or type 2 diabetes have an increased number of smaller mitochondrial compared with lean individuals [17, 44], supporting at least part of our findings.

It has been shown that an alteration in the balance between nuclear and mtDNA-encoded OXPHOS complexes, the so-called mitonuclear protein imbalance, is a stressor to mitochondria [32]. This proteotoxic stress can lead to the activation of the mtUPR, a specific mitochondrial stress response pathway mainly mediated by HSP60 [18]. In a study in *c elegans*, Houtkooper et al [32] pharmacologically induced a mitonuclear protein imbalance and showed that the activation of the mtUPR led to an increase in longevity in worms, suggesting that an activation of the mtUPR is beneficial for cellular function and organism longevity. Interestingly, we found in the present study that the mitonuclear protein balance, the ratio between a nuclear-encoded (COX4I1) and an mtDNA-encoded subunit (COX II) of complex IV, differed between lean individuals and endurance-trained athletes vs obese and type 2 diabetic participants. In addition, protein levels of HSP60, a marker of the mitochondrial stress response, were higher in athletes compared with obese and type 2 diabetic participants and correlated to the mitonuclear protein balance. The classical mitophagy pathway did not seem to be altered in our participant groups. So far, very little is known about the mtUPR in relation to oxidative capacity and insulin sensitivity in humans, although it was reported that protein expression of the mitochondrial chaperone HSP60 was elevated in skeletal muscle following a 3-month aerobic exercise intervention [45]. This change in HSP60 was associated with an induction of PGC-1α and mitochondrial DNA copy number [45], suggesting a positive relation between these events. In relation to lifelong training, it has also been reported that mitochondrial turnover is a necessity to maintain mitochondrial quality in muscle, requiring a balance between mitochondrial biogenesis, fusion and fission [46]. Together, these results suggest that with exercise training a high mitochondrial turnover induces beneficial mitochondrial stress responses that may in turn promote oxidative capacity and insulin sensitivity. Although it is tempting to speculate that the reverse may induce insulin resistance and type 2 diabetes, this cannot be deduced from the available data.

The current study presents some limitations. First of all, not all groups were similar in age and BMI and it is therefore difficult to delineate the contributions of both age and BMI to the differences observed in the comparisons between the athletes and lean individuals vs the obese and type 2 diabetic individuals. Furthermore, our data seem to indicate that primarily the endurance-trained individuals are different from all the other groups while lean, obese and type 2 diabetic patients often present similar values on the various read-outs. Nonetheless, inclusion of participants groups across the entire metabolic health spectrum facilitated the correlative analysis between the expression of the mitochondrial dynamics proteins and phenotypic characteristics related to oxidative capacity and insulin sensitivity. In addition, imaging analysis was performed on six individuals per group and limited to both extreme ends of the metabolic spectrum, i.e. the athletes vs the type 2 diabetes patients; this limits the extrapolation of the findings on mitochondrial network morphology to the healthy lean and obese individuals. Finally, we have chosen to normalise protein expression to total protein rather than mitochondrial protein. While it could be argued that differences in proteins such as OPA1, which have a mitochondrial targeting sequence, may relate to mitochondrial density, and therefore the increased expression of OPA1 in athletes may reflect increased mitochondrial fusion associated with increased mitochondrial biogenesis, any relationship between mitochondrial density and expression of mitochondrial dynamics regulatory proteins has not yet been established. Indeed, the majority of proteins involved in mitochondrial dynamics do not contain a mitochondrial targeting sequence and are often found in higher abundance outside the mitochondria [12]. Nevertheless, a relationship between mitochondrial quantity and the expression of mitochondrial network regulating proteins cannot be excluded at this time. Finally, our estimations of mitochondrial content (VDAC1, OXPHOS proteins) may be less accurate than other methods, such as electron microscopy or mtDNA copy number.

In conclusion, we here report that levels of OPA1, FIS1 and HSP60 are altered at the ends of the metabolic health spectrum. i.e. in individuals with type 2 diabetes and endurance-trained athletes, and that these proteins positively correlate to aerobic capacity and insulin sensitivity. Moreover, we demonstrated that these changes were accompanied by a more fragmented mitochondrial network morphology in diabetes patients vs endurance-trained athletes. Finally, lean individuals and athletes displayed a mitonuclear protein balance that was different from obese and type 2 diabetic individuals, which was accompanied by increased HSP60 protein levels in athletes, indicative of activation of the mtUPR. Together, our data show that various proteins involved in regulating mitochondrial dynamics and quality control relate to oxidative capacity and muscle insulin sensitivity in humans. Further studies should reveal whether mitochondrial dynamics regulatory proteins emerge as targets for the improvement of muscle mitochondrial capacity and insulin sensitivity.

## Supplementary information

ESM (PDF 1540 kb)

## Data Availability

The datasets generated during and/or analysed during the current study are available from the corresponding author on reasonable request.

## Abbreviations

COX II: Cytochrome C oxidase subunit II
COX4I1: Cytochrome C Oxidase Subunit 4I1
DRP1: Dynamin-related protein 1
FFM: Fat-free mass
FIS1: Fission protein 1
GIR: Glucose infusion rate
HSP60: Heat shock protein 60
LC3B: Microtubule-associated protein light chain 3B
MFN1: Mitofusin protein 1
MFN2: Mitofusin protein 2
mtDNA: Mitochondrial DNA
mtUPR: Mitochondrial unfolded protein response
OPA1: Optic atrophy protein 1
OXPHOS: Oxidative phosphorylation
PINK1: Phosphatase and tensin homologue (PTEN)-induced putative kinase 1
TOMM20: Translocase of outer mitochondrial membrane 20
VDAC1: Voltage-dependent anion-selective channel 1
