# Supplementary material for: Human skeletal muscle mitochondrial dynamics in relation to oxidative capacity and insulin sensitivity
Source: Diabetologia. 2020 Nov 30;64(2):424–36. doi: 10.1007/s00125-020-05335-w (PMC7801361; doi:10.1007/s00125-020-05335-w)
Supplement: Supplementary file 1 — (PDF 1540 kb) [file 125_2020_5335_MOESM1_ESM.pdf]

ELECTRONIC SUPPLEMENTARY MATERIAL

ESM figure 1

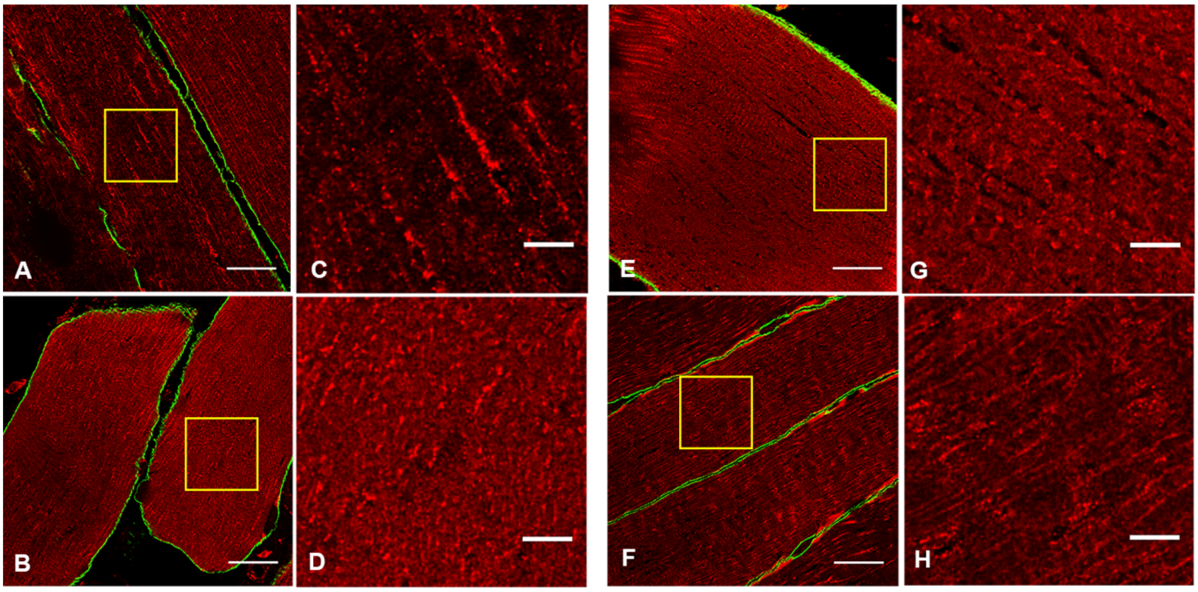

Representative confocal images of the mitochondrial network in type II muscle fibers. The cellular membrane was stained in green using laminin as a marker, in red the mitochondrial network is visualized using TOMM20 as a marker. In the overview images (A, B and E, F) scale bar, 20 µm; in the zoomed images (C, D and G, H) scale bar, 5 µm. (A-D) Representative overview and zoomed images of two separate individuals with type 2 diabetes. (E-H) Representative overview and zoomed images of two individual trained athletes

ESM figure 2

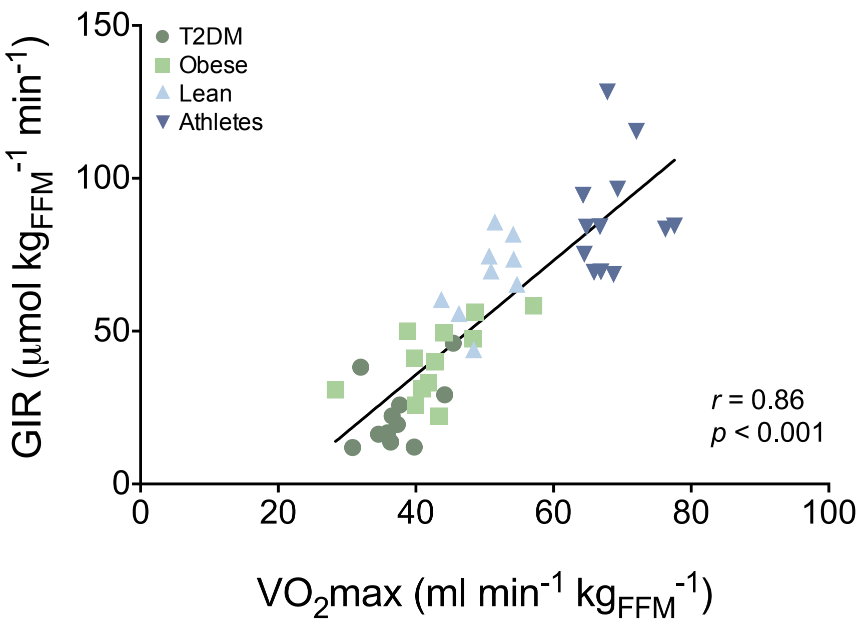

Association between maximal aerobic capacity and insulin sensitivity. Aerobic capacity is reflected by the maximum rate of oxygen consumption measured during an incremental exercise test ($\dot{V}O_{2max}$) and insulin sensitivity by the glucose infusion rate (GIR) during a hyperinsulinemic-euglycemic clamp. Correlations are computed with Pearson's *r*
